# Exploring factors in fear of COVID-19 and its GIS-based nationwide distribution: the case of Bangladesh

**DOI:** 10.1192/bjo.2021.984

**Published:** 2021-08-19

**Authors:** Mohammed A. Mamun

**Affiliations:** Director, CHINTA Research Bangladesh, Savar, Dhaka, Bangladesh, and Department of Public Health and Informatics, Jahangirnagar University, Savar, Dhaka, Bangladesh

**Keywords:** COVID-19 fear, psychological impact, fear of infection, fear of COVID-19 scale, GIS-based distribution, Bangladesh

## Abstract

**Background:**

The COVID-19 pandemic is a public health threat of international concern, intensifying peoples’ psychological risk and vulnerability by strengthening mental health stressors such as fear, panic and uncertainty. The unexpected fear of COVID-19 has been reported to be associated with suicide occurrences, similar to prior pandemics.

**Aims:**

Identifying the factors associated with fear of COVID-19 could help us to develop better mental health strategy and practice to improve the situation here in Bangladesh. This was the first attempt to present a Geographic Information System (GIS)-based distribution of fear of COVID-19 across the country's administrative districts in a nationwide sample.

**Method:**

Data for a total of 10 067 individuals were collected by an online survey during the first wave of the pandemic (1 to 10 April 2020); data for 10 052 participants were finally analysed after excluding 15 transgender individuals. The survey questionnaire included items concerning sociodemographic, behavioural and health-related variables, COVID-19-related issues, and the Bangla Fear of COVID-19 Scale.

**Results:**

The mean fear of COVID-19 scores was 21.30 ± 6.01 (out of a possible 35) in the present sample. Female gender, highly educated, non-smoker, non-alcohol consumer, having chronic diseases, using social media, and using social media and not using newspapers as COVID-19 information sources were associated with a higher level of fear of COVID-19. Higher levels of fear of COVID-19 were found in districts of Magura, Panchagarh, Tangail, Sunamganj and Munshiganj; by contrast, Kushtia, Pirojpur, Chapainawabganj, Jhalokathi and Naogaon districts had lower fear of COVID-19. Based on the GIS-distribution, fear of COVID-19 was significantly associated with the district as well as in respect to its gender-based and education-level-based associations. However, fear of COVID-19 and COVID-19 cases were heterogeneously distributed across the districts; that is, no consistent association of higher COVID-19 cases with higher fear of COVID-19 was found.

**Conclusions:**

This study being exploratory in nature may help to facilitate further studies, as well as directing governmental initiatives for reducing fear of COVID-19 in at-risk individuals. Providing adequate resources and mental health services in the administrative regions identified as highly vulnerable to fear of COVID-19 is recommended.

## Introduction

During viral outbreaks such as the COVID-19 pandemic, the perceived risk of infection with the virus increases emotional issues including fear, panic, worries and uncertainties.^1^ The effects of fear of COVID-19 on psychological health were visualised during the early period of the pandemic in Bangladesh.^2^ The first COVID-19-related suicide case occurred on 25 March 2020, with an allegation of fear of COVID-19 as the suicide stressor;^3^ whereas the first COVID-19 patient was diagnosed on 8 March 2020.^4^ Extreme psychological effects (such as suicide) related to fear of COVID-19 have not only been observed in Bangladesh; several case-series studies from other countries have also been reported.^5,6^ Similarly, fear of infection was regarded as a prominent suicide factor in prior pandemics.^7,8^ Studies have confirmed that fear of COVID-19 acts as a mediator to increase psychological events such as depression and anxiety,^2^ thereby making individuals psychologically vulnerable to suicidality.^9^ Thus, increments in suicide rates have been seen owing to burdens related to fear of COVID-19, as well as other devastating effects of the pandemic (see Mamun^9^ a systematic review of studies on suicide in Bangladesh).

For understanding the effects of fear of COVID-19 on people and adopting effective mental health strategies, there is a need for epidemiological studies.^10,11^ However, there has been a lack of studies assessing fear of COVID-19 compared with other aspects of mental suffering, including depression, anxiety and stress, in Bangladesh.^11^ To the best of the author's knowledge, three studies have assessed risk factors for fear of COVID-19 across different cohorts (i.e., the general population,^12^ the general population and healthcare professionals,^4^ and older adults^13^), whereas another study explored its mediating role in career anxiety among students.^2^ However, none of these studies considered such a large sample as that used in the present study, provided any predictive models explaining the fear of COVID-19, or considered its nationwide spatial distribution.

More engagement with COVID-19-preventive behaviours is frequently observed in individuals with higher COVID-19 fear,^14^ where fear of COVID-19 acts as a motivational factor.^15^ Whereas, practice of behaviours related to COVID-19 knowledge, fear and so on, increase among female individuals and those with higher education levels.^16–18^ However, none of the aforementioned Bangladeshi studies considered the associations of education level or gender with fear of COVID-19; these associations are investigated herein.

## Method

### Study procedure, participants and ethics

The present nationwide cross-sectional study was conducted during the early stage of the COVID-19 pandemic, between 1 and 10 April 2020. The survey was conducted via online promotion; three or four research assistants from each of the 64 districts in Bangladesh were employed to facilitate access to the survey link through their social media platforms. The research team approached nearly 11 000 participants; 10 067 were enrolled in the present study after meeting the criteria of being Bangladeshi residents aged over ten years. Ethical approval for this project was obtained from two Bangladeshi institutes (see Ethics statement). For detailed information about the study sample, procedures, participants, ethics and data access, please refer to the following publications.^19–23^

### Measures

#### Independent variable

Information related to basic sociodemographic variables including age, gender, education level, occupation status, marital status, current residence type and district of residence was collected in this study. Education level was categorised into two types: lower education (up to secondary high school) and higher education (tertiary education). Behaviour- and health-related variables (smoking status, perceived health status [based on a Likert scale], social media use, etc.) were also included in this study. Participants were asked whether anyone had returned to their home from Dhaka or outside the Bangladesh area/country which were highly affected by COVID-19. Finally, sources of COVID-19-related information (social media, newspaper, television, YouTube, etc.) were also assessed.

#### Dependent variable

Fear of COVID-19 was assessed using the Bangla Fear of COVID-19 Scale,^23^ which was considered the dependent variable. Bangla Fear of COVID-19 Scale was adapted and validated from the English version of the scale originally developed by Ahorsu et al.^24^ The screening tool comprises a total of seven items (e.g., ‘I am afraid of losing my life because of coronavirus-19’), and responses are recorded on a five-point Likert scale (1 = strongly disagree to 5 = strongly agree). The score ranges between 7 to 35, where a higher score indicates greater fear of COVID-19. In the present study, Cronbach's alpha was very good (0.88).

### Statistical analysis

Of the 10 067 respondents, data for 10 052 participants were finally analysed after exclusion of 15 transgender individuals (because of a low response rate). Data were analysed using SPSS 23.0 for descriptive statistics and regression models. Independent *t*-tests and analysis of variance were performed to determine the associations between the studied variables and fear of COVID-19 (as a dependent variable). Hierarchical linear regression was used to observe the independent variables’ contributions to fear of COVID-19. The normality of the distributions (e.g., skewness and kurtosis values) and multicollinearity (e.g., variance inflation factor and tolerance values) were also tested, and no issues were observed. *P* < 0.05 was set as statistically significant with 95% confidence intervals. Also, gender-based and education-level-based *post hoc* analyses were performed for all tests. Finally, ArcGIS 10.8 was used to examine the nationwide distribution of fear of COVID-19 and its relationship with COVID-19 cases, gender and education level across districts.

### Ethics statement

Ethical permission to conduct this study was granted by the Biosafety, Biosecurity and Ethical Committee of Jahangirnagar University, Bangladesh (BBEC, JU/M 2O20/COVlD-l9/(9)2), and the Institute of Allergy and Clinical Immunology of Bangladesh ethics board, Bangladesh (IRBIACIB/CEC/03202005).

### Consent statement

All participants provided online informed consent prior to starting the survey. They were informed about the purpose and nature of the study, and they had the right to withdraw their data if they wanted to. For participants less than 18 years old, parental consent was obtained. All participants were assured about the confidentiality of their data.

## Results

### Characteristics of the participants

Of the total of 10 052 participants, the majority were male (56.2%, *n* = 5650), highly educated (80.8%, *n* = 8127), students (58.4%) and single with respect to marital status (70.3%), and 40.1% resided in the divisional city. About 71.3% were aged 20 to 29 years, and the mean age of the total sample was 26.95 ± 9.632 years (Table 1). About 14.7% were smokers, 2.6% reported consuming alcohol, 24.6% had a chronic disease and 90.9% used social media (Table 2). Approximately 12.9% and 2.5% of the participants reported that someone had returned to their home from the COVID-19 high-risk region Dhaka and from a foreign country, respectively. Of the COVID-19 information sources, social media was prominently reported as being used (Table 3).

**Table 1:** Distribution of fear of COVID-19 scores across sociodemographic variables

Study variables	Total sample		Gender		Education	
	*n*, %	Mean ± s.d.	Male	Female	Lower	Higher
Age, years						
10–19	683, 6.8%	20.46 ± 5.95	19.67 ± 5.85	21.19 ± 5.95	19.82 ± 6.16	21.58 ± 5.39
20–29	7165, 71.3%	21.47 ± 5.86	20.33 ± 5.89	22.91 ± 5.50	20.93 ± 6.05	21.53 ± 5.84
30–39	1218, 12.1%	21.34 ± 6.34	20.19 ± 6.13	23.38 ± 6.18	20.98 ± 7.54	21.42 ± 6.05
40–49	410, 4.1%	21.09 ± 6.42	20.50 ± 6.26	21.58 ± 6.53	21.08 ± 6.55	21.10 ± 6.27
50–59	371, 3.7%	20.37 ± 6.33	20.51 ± 6.08	20.17 ± 6.66	20.66 ± 6.88	19.95 ± 5.43
≥60	196, 1.9%	19.88 ± 6.98	19.58 ± 7.01	20.61 ± 6.90	19.55 ± 7.05	20.40 ± 6.89
***P*-value**	−	<0.001	0.278	<0.001	0.022	0.014
Occupation						
Unemployed	360, 3.6%	21.42 ± 6.32	20.89 ± 6.33	22.52 ± 6.19	19.23 ± 7.11	21.87 ± 6.07
Employed	2586, 25.7%	21.01 ± 6.25	20.26 ± 6.18	23.25 ± 5.92	20.37 ± 7.03	21.17 ± 6.04
Retired	91, 0.9%	20.53 ± 6.74	20.44 ± 6.78	21.08 ± 6.71	21.15 ± 5.93	20.26 ± 7.08
Housewife	1147, 11.4%	21.83 ± 6.41	20.29 ± 5.23	22.19 ± 6.61	21.26 ± 6.86	22.24 ± 6.02
Student	5868, 58.4%	21.33 ± 5.77	20.22 ± 5.85	22.64 ± 5.39	20.39 ± 5.88	21.48 ± 5.74
***P*-value**	−	0.003	0.563	0.008	0.051	<0.001
Residence						
Village	2330, 23.2%	20.89 ± 6.38	20.41 ± 6.22	21.97 ± 6.59	20.01 ± 7.17	21.27 ± 5.96
Upazila town	1358, 13.5%	21.57 ± 5.85	20.69 ± 5.91	22.94 ± 5.49	21.06 ± 6.00	21.69 ± 5.81
District town	2330, 23.2%	21.56 ± 5.91	20.32 ± 5.95	22.81 ± 5.59	21.05 ± 6.12	21.68 ± 5.85
Divisional town	4034, 40.1%	21.29 ± 5.88	19.96 ± 5.82	22.67 ± 5.63	20.71 ± 6.02	21.38 ± 5.86
***P*-value**	−	<0.001	0.014	0.007	0.023	0.084
Administrative division						
Barishal	207, 2.1%	21.49 ± 6.91	20.05 ± 7.32	23.65 ± 5.64	23.00 ± 8.15	21.17 ± 6.61
Chittagong	2406, 23.9%	21.38 ± 5.94	20.40 ± 5.93	22.74 ± 5.69	21.24 ± 6.39	21.42 ± 5.83
Dhaka	4286, 42.6%	21.53 ± 5.81	20.28 ± 5.76	22.92 ± 5.54	20.94 ± 5.82	21.64 ± 5.80
Khulna	1044, 10.4%	20.62 ± 6.52	19.85 ± 6.50	21.62 ± 6.41	18.67 ± 7.35	21.43 ± 5.96
Mymensingh	256, 2.5%	21.37 ± 6.06	20.10 ± 6.15	23.33 ± 5.40	21.60 ± 5.28	21.33 ± 6.21
Rajshahi	944, 9.4%	21.11 ± 6.08	20.52 ± 5.97	22.04 ± 6.15	20.94 ± 6.71	21.16 ± 5.90
Rangpur	558, 5.6%	20.42 ± 6.09	19.99 ± 5.91	21.31 ± 6.37	18.47 ± 6.49	21.06 ± 5.82
Sylhet	333, 3.3%	21.55 ± 5.99	20.32 ± 6.19	22.92 ± 5.46	22.24 ± 5.83	21.40 ± 6.02
***P*-value**	−	<0.001	0.576	<0.001	<0.001	0.345
Marital status						
Single	7071, 70.3%	21.20 ± 5.79	20.23 ± 5.83	22.62 ± 5.44	20.43 ± 6.04	21.32 ± 5.74
Married	2838, 28.2%	21.56 ± 6.46	20.40 ± 6.37	22.74 ± 6.35	20.82 ± 6.92	21.88 ± 6.24
Divorced/widowed/other	143, 1.4%	20.90 ± 6.80	19.78 ± 7.35	21.35 ± 6.56	19.93 ± 7.27	22.37 ± 5.79
***P*-value**	−	0.016	0.571	0.064	0.284	0.001

**Table 2:** Distribution of fear of COVID-19 scores across behaviour- and health-related variables

Study variables	Total sample		Gender		Education	
	*n*, %	Mean ± s.d.	Male	Female	Lower	Higher
Current smoker						
Yes	1478,14.7%	20.29 ± 6.18	20.20 ± 6.17	22.98 ± 6.32	19.94 ± 6.63	20.39 ± 6.07
No	8574,85.3%	21.47 ± 5.95	20.29 ± 5.92	22.62 ± 5.77	20.70 ± 6.47	21.65 ± 5.82
***P*-value**	−	<0.001	0.612	0.659	0.059	<0.001
Alcohol use						
Yes	264, 2.6%	19.85 ± 6.35	19.62 ± 6.32	22.29 ± 6.27	18.78 ± 6.84	20.23 ± 6.15
No	9788, 97.4%	21.33 ± 5.99	20.29 ± 5.96	22.63 ± 5.77	20.64 ± 6.48	21.50 ± 5.86
***P*-value**	−	<0.001	0.085	0.776	0.021	0.003
Perceived health status						
Very good	6899, 68.6%	20.96 ± 6.02	19.94 ± 5.94	22.37 ± 5.86	20.05 ± 6.62	21.14 ± 5.88
Acceptable	2807, 27.9%	22.06 ± 5.83	21.04 ± 5.92	23.19 ± 5.53	21.29 ± 6.20	22.30 ± 5.69
Poor	312, 3.1%	21.85 ± 6.34	21.16 ± 6.61	22.56 ± 5.99	21.67 ± 6.28	21.97 ± 6.39
Very poor	34, 0.3%	21.50 ± 7.25	20.94 ± 8.08	22.12 ± 6.39	21.09 ± 8.32	21.69 ± 6.87
***P*-value**	−	<0.001	<0.001	<0.001	<0.001	<0.001
Comorbidity status						
No	7581, 75.4%	21.15 ± 5.96	20.04 ± 5.94	22.61 ± 5.68	20.17 ± 6.57	21.34 ± 5.82
Yes	2471, 24.6%	21.75 ± 6.10	20.99 ± 6.06	22.67 ± 6.04	21.31 ± 6.30	21.92 ± 6.03
***P*-value**	−	<0.001	<0.001	<0.001	<0.001	<0.001
Social media use						
Yes	9139, 90.9%	21.40 ± 5.87	20.29 ± 5.88	22.88 ± 5.52	20.90 ± 6.00	21.48 ± 5.85
No	913, 9.1%	20.26 ± 7.13	19.91 ± 7.02	20.58 ± 7.22	20.01 ± 7.26	21.06 ± 6.64
***P*-value**	−	<0.001	0.196	<0.001	0.004	0.302
Social media use frequency						
More than 4 days per week	292, 2.9%	21.51 ± 6.18	20.50 ± 6.27	23.08 ± 5.72	22.42 ± 6.39	21.25 ± 6.11
2–3 days per week	317, 3.2%	20.99 ± 5.56	20.05 ± 5.95	22.18 ± 4.79	22.17 ± 5.73	20.41 ± 5.40
Every day	4077, 40.6%	21.42 ± 5.83	20.40 ± 5.84	22.95 ± 5.47	20.63 ± 5.94	21.55 ± 5.80
Several times per day	4443, 44.2%	21.45 ± 5.92	20.28 ± 5.90	22.86 ± 5.63	20.82 ± 6.07	21.54 ± 5.89
***P*-value**	−	0.583	0.764	0.439	0.02	0.04

**Table 3:** Distribution of fear of COVID-19 scores across the COVID-19-related variables

Study variables	Total sample		Gender		Education	
	*n*, %	Mean ± s.d.	Male	Female	Lower	Higher
Someone returns home from Dhaka						
Yes	1292, 12.9%	21.14 ± 6.01	20.15 ± 5.99	22.62 ± 5.72	20.82 ± 6.66	21.22 ± 5.83
No	7660, 76.2%	21.37 ± 5.99	20.36 ± 5.98	22.66 ± 5.78	20.55 ± 6.48	21.57 ± 5.86
***P*-value**	−	0.208	0.380	0.870	0.540	0.074
Someone returns home from a COVID-19-affected country						
Yes	254, 2.5%	21.17 ± 6.06	19.94 ± 5.76	22.59 ± 6.11	20.05 ± 6.38	21.49 ± 5.94
No	9798, 97.5%	21.30 ± 6.01	20.27 ± 5.98	22.63 ± 5.77	20.59 ± 6.50	21.47 ± 5.87
***P*-value**	−	0.733	0.520	0.951	0.538	0.962
Source of COVID-19 information: social media						
No	1784, 17.7%	20.27 ± 6.55	19.36 ± 6.49	21.27 ± 6.47	20.19 ± 7.06	20.33 ± 6.05
Yes	8268, 82.3%	21.52 ± 5.86	20.45 ± 5.86	22.95 ± 5.55	20.89 ± 5.99	21.62 ± 5.83
***P*-value**	−	<0.001	<0.001	<0.001	0.02	<0.001
Source of COVID-19 information: YouTube						
No	2755, 27.4%	21.32 ± 6.02	20.22 ± 5.97	22.45 ± 5.86	20.65 ± 6.59	21.52 ± 5.82
Yes	7297, 72.6%	21.27 ± 5.99	20.32 ± 5.99	22.94 ± 5.61	20.43 ± 6.27	21.41 ± 5.93
***P*-value**	−	0.721	0.548	0.007	0.493	0.411
Source of COVID-19 information: newspaper						
No	5124, 51.0%	21.46 ± 6.04	20.31 ± 6.04	22.65 ± 5.81	20.64 ± 6.64	21.72 ± 5.82
Yes	4928, 49.0%	21.13 ± 5.97	20.23 ± 5.93	22.58 ± 5.73	20.48 ± 6.26	21.24 ± 5.91
***P*-value**	−	0.005	0.574	0.688	0.593	<0.001
Source of COVID-19 information: television						
No	2755, 27.4%	21.24 ± 6.01	20.16 ± 5.99	22.65 ± 5.72	20.37 ± 6.43	21.40 ± 5.91
Yes	7297, 72.6%	21.32 ± 6.01	20.31 ± 5.97	22.62 ± 5.79	20.64 ± 6.52	21.50 ± 5.86
***P*-value**	−	0.531	0.396	0.863	0.451	0.514
Source of COVID-19 information: health website						
No	5559, 55.3%	21.35 ± 6.06	20.32 ± 6.04	22.55 ± 5.86	20.58 ± 6.69	21.63 ± 5.79
Yes	4493, 44.7%	21.24 ± 5.94	20.20 ± 5.91	22.73 ± 5.65	20.56 ± 5.80	21.31 ± 5.95
***P*-value**	−	0.330	0.440	0.292	0.948	0.014
Source of COVID-19 information: other sources						
No	8106, 80.6%	21.46 ± 5.92	20.34 ± 5.92	22.79 ± 5.65	20.78 ± 6.42	21.61 ± 5.79
Yes	1946, 19.4%	20.63 ± 6.29	20.03 ± 6.18	21.75 ± 6.34	19.93 ± 6.69	20.85 ± 6.15
***P*-value**	−	<0.001	0.106	<0.001	0.015	<0.001

### Distribution of fear of COVID-19 scores

The overall mean score for fear of COVID-19 was 21.30 ± 6.01. The distribution of COVID-19 fear scores across sociodemographic variables is presented in Table 1; all of the associations were statistically significant. Female participants had higher fear of COVID-19 scores compared with their male counterparts (22.62 ± 5.78 *v*. 20.27 ± 5.98; *t* = −19.913, *P* < 0.001), and highly educated participants reported higher fear of COVID-19 than less educated ones (21.47 ± 5.87 *v*. 20.58 ± 6.50; *t* = −5.856, *P* < 0.001). Participants being smokers and consuming alcohol had lower scores for COVID-19 fear, whereas higher scores were found for participants with chronic diseases and those who were social media users (Table 2). Of the COVID-19-related variables, participants who used social media as a source of COVID-19 information had higher fear of COVID-19 scores (21.52 ± 5.86 *v*. 20.27 ± 6.55; *P* < 0.001), whereas lower scores were found for those who read newspapers for COVID-19 information (21.13 ± 5.97 *v*. 21.46 ± 6.04; *P* = 0.005) (Table 3). Gender-based and education-level-based distributions of fear of COVID-19 scores are presented in Tables 1–3.

### Predictive factors of fear of COVID-19

The results of the hierarchical linear regression analysis of factors predicting fear of COVID-19 are presented in Table 4 (total sample), Supplementary Table 1 available at https://doi.org/10.1192/bjo.2021.984 (gender-based) and Supplementary Table 2 (education-level-based). However, sociodemographic variables were included in model 1, whereas in model 2, behaviour- and health-related factors were compiled with sociodemographic variables. Finally, COVID-19-related variables were included in model 3 with the variables entered in model 2. All of the models were statistically significant. A 5.1% variance of fear of COVID-19 scores was predicted in the total sample; this increased to 5.7% and 6.2% in model 2 and model 3, respectively. The gender-based and education-level-based explanatory power of fear of COVID-19 can be found in Supplementary Tables 1 and 2, respectively. The final gender-based hierarchical regression model explained 2% and 1.9% of the variance in predicted fear of COVID-19 for males and females, respectively (Supplementary Table 1), whereas 4.7% and 6.7% of the variance were explained in the final education-level model for lower and higher education levels, respectively (Supplementary Table 2).

**Table 4:** Hierarchical regression analysis of factors predicting fear of COVID-19

Models		B	s.e.	*β*	*t*	*P*
**Model 1** *R*^2^ = 0.051, Adjusted *R*^2^ = 0.050, *P* < 0.001	(Constant)	18.071	0.604		29.903	<0.001
	Age	−0.026	0.013	−0.030	−2.022	0.043
	Gender^a^	2.594	0.136	0.219	19.138	<0.001
	Education^b^	0.526	0.185	0.031	2.843	0.004
	Occupation^c^	−0.099	0.056	−0.023	−1.758	0.079
	Residence^d^	−0.215	0.056	−0.043	−3.838	<0.001
	Administrative division^e^	−0.081	0.037	−0.024	−2.196	0.028
	Marital status^f^	0.542	0.186	0.041	2.907	0.004
**Model 2** *R*^2^ = 0.057, Adjusted *R*^2^ = 0.056, *P*<0.001	(constant)	15.645	1.261		12.411	<0.001
	Age	−0.039	0.013	−0.046	−3.001	0.003
	Gender^a^	2.499	0.142	0.211	17.567	<0.001
	Education^b^	0.659	0.186	0.039	3.540	<0.001
	Occupation^c^	−0.118	0.056	−0.028	−2.101	0.036
	Residence^d^	−0.206	0.056	−0.042	−3.663	<0.001
	Administrative division^e^	−0.080	0.037	−0.024	−2.172	0.030
	Marital status^f^	0.529	0.186	0.040	2.843	0.004
	Smoking status^g^	0.188	0.205	0.011	0.916	0.359
	Alcohol consumption^g^	0.379	0.431	0.010	0.879	0.380
	Perceived health status^h^	0.393	0.067	0.065	5.836	<0.001
	Comorbidity status^i^	0.458	0.162	0.033	2.832	0.005
	Social media user^g^	1.153	0.654	0.019	1.764	0.078
	Social media use frequency^j^	−0.085	0.090	−0.010	−0.939	0.348
**Model 3** *R*^2^ = 0.062, Adjusted *R*^2^ = 0.059, *P* < 0.001	(constant)	14.428	1.529		9.438	<0.001
	Age	−0.037	0.013	−0.043	−2.820	0.005
	Gender^a^	2.452	0.144	0.207	17.063	<0.001
	Education^b^	0.655	0.187	0.039	3.493	<0.001
	Occupation^c^	−0.120	0.056	−0.028	−2.128	0.033
	Residence^d^	−0.201	0.057	−0.041	−3.516	<0.001
	Administrative division^e^	−0.072	0.037	−0.021	−1.957	0.050
	Marital status^f^	0.493	0.186	0.037	2.645	0.008
	Smoking status^g^	0.174	0.205	0.010	0.848	0.396
	Alcohol consumption^g^	0.354	0.431	0.009	0.820	0.412
	Perceived health status^h^	0.386	0.067	0.064	5.729	<0.001
	Comorbidity status^i^	0.485	0.162	0.035	2.996	0.003
	Social media user^g^	1.387	0.655	0.023	2.119	0.034
	Social media use frequency^j^	−0.126	0.091	−0.015	−1.382	0.167
	SRHF Dhaka^g^	0.324	0.185	0.019	1.754	0.080
	SRHF COVID-19-infected country^g^	−0.124	0.409	−0.003	−0.302	0.763
	SCI: social media^i^	1.056	0.213	0.056	4.946	<0.001
	SCI: YouTube^i^	0.095	0.140	0.008	0.677	0.498
	SCI: Newspaper^i^	−0.271	0.145	−0.023	−1.868	0.062
	SCI: television^i^	0.189	0.153	0.015	1.232	0.218
	SCI: health website^i^	−0.087	0.137	−0.007	−0.634	0.526
	SCI: other sources^i^	−0.400	0.173	−0.026	−2.313	0.021

B, unstandardised regression coefficient; β, standardised regression coefficient; SBHF, someone has returned home from; SCI, source of COVID-19 information.

a. 1 = Male, 2 = Female.

b. 1 = Lower, 2 = Higher.

c. 1 = Unemployed, 2 = Employed, 3 = Retried, 4 = Housewife, 5 = Student.

d. 1 = Village, 2 = Upzilla town, 3 = District-level town, 4 = Divisional city.

e. 1 = Barishal, 2 = Chittagong, 3 = Dhaka, 4 = Khulna, 5 = Mymensingh, 6 = Rajshahi, 7 = Rangpur, 8 = Sylhet.

f. 1 = Single, 2 = Married, 3 = Divorced/widowed/other.

g. 1 = Yes, 2 = No.

h. 1 = Very good, 2 = Acceptable, 3 = Poor, 4 = Very poor.

i. 1 = No, 2 = Yes.

j. 1 = More than 4 days per week, 2 = 2–3 days per week, 3 = every day, 4 = several times per day.

### District-wise distribution of fear of COVID-19

**Fig. 1:**
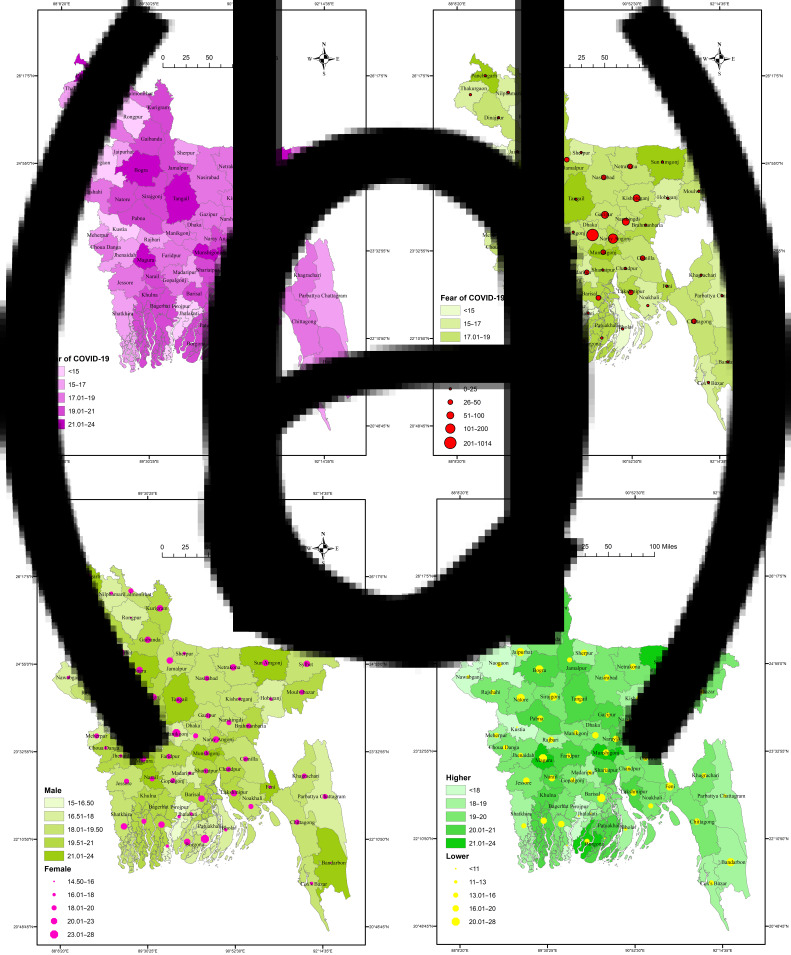
(a) Distribution of fear of COVID-19 across districts in Bangladesh. (b) Distribution of fear of COVID-19 and COVID-19 cases across districts. (c) Gender-based distribution of fear of COVID-19 across districts. (d) Education-level-based distribution of fear of COVID-19 across districts.

District-wise spatial distribution of fear of COVID-19 is presented in Fig. 1(a) and was statistically significant (*F* = 5.897, *P* < 0.001). Higher levels of fear of COVID-19 were found in Magura, Panchagarh, Tangail, Sunamganj, Munshiganj, Bogra, Barguna, Narail and Naraynganj; by contrast, Kushtia, Pirojpur, Chapainawabganj, Jhalokathi, Naogaon and Rangpur districts had lower fear of COVID-19.

### District-wise relationship of fear of COVID-19 with COVID-19 cases

Figure 1(b) represents the relationship of fear of COVID-19 with numbers of COVID-19 cases in the respective districts. However, fear of COVID-19 and COVID-19 cases were heterogeneously distributed across the districts; that is, no consistent association of higher COVID-19 cases with higher fear of COVID-19 was found.

### District-wise distribution of gender-based fear of COVID-19

The gender-based distribution of fear of COVID-19 across districts is presented in Fig. 1(c); the associations of both genders with fear of COVID-19 were found to be statistically significant (*F* = 2.932, *P* < 0.001, and *F* = 4.415, *P* < 0.001 for males and females, respectively). Male participants from the districts of Magura, Munshiganj, Sunamganj, Bandarban, Feni, Tangail, Panchagarh and Bogra had higher fear of COVID-19 scores, as did female participants from Patuakhali, Joypurhat, Manikganj, Lakshmipur, Panchagarh,Barishal, Tangail and Barguna. However, both genders from the districts of Panchagarh, Bogra, Tangail and Sunamganj reported higher fear of COVID-19.

### District-wise distribution of education-level-based fear of COVID-19

The education-level-based distribution of fear of COVID-19 across districts is presented in Fig. 1(d); the associations of both education levels with fear of COVID-19 were found to be statistically significant (*F* = 6.226, *P* < 0.001, and *F* = 1.773, *P* < 0.001 for lower education and higher education, respectively). Participants with lower education from the districts of Lalmonirhat, Barishal, Bandarban, Tangail, Sirajganj, Magura, Bogra, Natore and Gaibandha had higher fear of COVID-19 scores, compared with those from Sunamganj, Panchagarh, Magura, Munshiganj, Barguna Naraynganj, Tangail, Kurigram and Shariatpur for more highly educated participants. However, participants of both education levels from the districts of Tangail, Magura, Bogra and Gaibandha had higher fear of COVID-19 scores.

## Discussion

The COVID-19 pandemic is a public health threat that can increase peoples’ psychological risk and vulnerability by intensifying mental health stressors including fear, panic and uncertainty. Furthermore, the unexpected fear of COVID-19 can be life-threatening by leading to a drastic decision such as suicide.^5–8^ Therefore, identifying the factors associated with fear of COVID-19 can help with the adoption and implementation of a better mental health strategy. Herein, factors related to fear of COVID-19 were identified from a nationwide sample collected by an online survey during the first wave of the pandemic in Bangladesh. The sample was limited owing to the use of an online survey with responses overwhelmingly from educated individuals; therefore, a *post hoc* analysis considering participants’ education levels was conducted. This was the first attempt to present a GIS-based distribution of fear of COVID-19 across the administrative districts of the country.

The overall mean fear of COVID-19 score was 21.30 ± 6.01 (out of a possible of 35) in the present sample. Use of the same instrument to assess levels of fear of COVID-19 has been reported in other Bangladeshi studies. For example, a mean score of 18.53 ± 5.013 was reported for a survey conducted from April to May 2020 in a sample of 2157 individuals,^12^ and 19.4 ± 6.1 in another study comprising a total of 1032 elderly individuals conducted in October 2020.^13^ Thus, the present sample showed higher fear of COVID-19 scores, although comparisons were limited because of the study implementation time and lack of representativeness of samples across these Bangladeshi studies.

The study found female gender to be a risk factor for reporting higher fear of COVID-19; similarly, gender was identified as one of the significant predictors of fear of COVID-19 in other Bangladeshi studies.^4,12,13^ Reckless and irresponsible attitudes towards COVID-19 are more frequently observed among male participants, indicating that females are more conscious of preventive COVID-19 behaviours, leading to higher fear of COVID-19.^16^ Similarly, the level of consciousness increases with higher levels of educational attainment. That is, more educated individuals are at risk of higher fear of COVID-19 owing to the avoidance behaviours they adopt to protect themselves from potential virus infection,^16–18^ which is also reported in the study.

The studied variables predicted the explanatory power of the fear of COVID-19 in this study. Initially, sociodemographic variables (i.e., age, gender, education, occupation, residence, administrative division and marital status) predicted a 5.1% variance in fear of COVID-19. When behaviour- and health-related factors (i.e., smoking status, alcohol consumption, perceived health status, comorbidity status, social media use and frequency of social media use) were included, together with sociodemographic variables, the variance in fear of COVID-19 increased to 5.7%. Finally, after adjusting for COVID-19-related variables, an additional 0.5% of explanatory power was obtained. As this was an exploratory study, comparisons of the present models with those of prior studies were limited. However, it is anticipated that these models will help to facilitate further investigations.

The GIS-based distribution-related all of the associations with fear of COVID-19 were statistically significant. Higher fear of COVID-19 was found in the districts of Magura, Panchagarh, Tangail, Sunamganj, Munshiganj, Bogra, Barguna, Narail and Naraynganj. However, with respect to the relationship between COVID-19 fear and numbers of COVID-19 cases across districts, a heterogeneous distribution was found; that is, there was no consistent relationship between higher or lower COVID-19 case numbers with higher or lower fear of COVID-19. The districts of Panchagarh, Bogra, Tangail and Sunamganj reported higher fear of COVID-19 for both genders, although there were distinct districts reporting higher COVID-19 fear for each gender. Similarly, the districts of Tangail, Magura, Bogra and Gaibandha had higher fear of COVID-19 scores for participants of both education levels.

Several methodological issues of the present study should be noted as limitations. First, it was a cross-sectional study; this may hinder the ability to infer causal associations from the current findings. Online surveys can be limited by selection bias (e.g., the overwhelming response from educated persons). Moreover, important behavioural predictors influencing the level of fear of COVID-19 (perceived behavioural control, action self-efficacy, etc.) were not considered in this study.^25^ However, despite these limitations, this study provides baseline information across districts on an important but unexplored issue, fear of COVID-19, in the context of Bangladesh.

## Data Availability

The data used in this study can be accessed at: https://doi.org/10.1016/j.dib.2020.106621.

## Supplementary material

For supplementary material accompanying this paper visit http://dx.doi.org/10.1192/bjo.2021.984. click here to view supplementary material
