# Supplementary material for: Exploring factors in fear of COVID-19 and its GIS-based nationwide distribution: the case of Bangladesh
Source: BJPsych Open. 2021 Aug 19;7(5):e150. doi: 10.1192/bjo.2021.984 (PMC8376996; doi:10.1192/bjo.2021.984)
Supplement: Supplementary file 1 [file S2056472421009844sup001.docx]

**Supplementary Table 1.** Gender-based hierarchical regression analysis predicting fear of COVID-19

| **Models** | | **Male** | | | **Female** | | |
| --- | --- | --- | --- | --- | --- | --- | --- |
|  |  | ***β*** | ***t*** | ***p*** | ***β*** | ***t*** | ***p*** |
| **Model 1**  Male: R^2^=.003, Adjusted R^2^=.002, *p*=0.015  Female: R^2^=.009, Adjusted R^2^=.007, *p*<0.001 | (Constant) |  | 27.380 | <.001 |  | 22.875 | <.001 |
|  | Age | -.029 | -1.417 | .157 | -.026 | -1.142 | .254 |
|  | Education level ^a^ | .011 | .766 | .444 | .063 | 3.643 | <.001 |
|  | Occupation ^b^ | -.023 | -1.237 | .216 | -.023 | -1.176 | .240 |
|  | Residence ^c^ | -.049 | -3.283 | .001 | -.033 | -1.880 | .060 |
|  | Administrative division ^d^ | -.010 | -.707 | .480 | -.045 | -2.612 | .009 |
|  | Marital status ^e^ | .033 | 1.686 | .092 | .050 | 2.279 | .023 |
| **Model 2**  Male: R^2^=.014, Adjusted R^2^=.012, *p*<0.001  Female: R^2^=.012, Adjusted R^2^=.009, *p*<0.001 | (Constant) |  | 11.677 | <.001 |  | 6.405 | <.001 |
|  | Age | -.048 | -2.312 | .021 | -.036 | -1.547 | .122 |
|  | Education level ^a^ | .022 | 1.461 | .144 | .067 | 3.881 | <.001 |
|  | Occupation ^b^ | -.027 | -1.492 | .136 | -.027 | -1.372 | .170 |
|  | Residence ^c^ | -.049 | -3.265 | .001 | -.029 | -1.678 | .093 |
|  | Administrative division ^d^ | -.009 | -.644 | .520 | -.045 | -2.608 | .009 |
|  | Marital status ^e^ | .030 | 1.545 | .123 | .051 | 2.306 | .021 |
|  | Smoking status ^f^ | .014 | .906 | .365 | .001 | .039 | .969 |
|  | Alcohol consuming ^f^ | .014 | .880 | .379 | .007 | .369 | .712 |
|  | Perceived health status ^g^ | .079 | 5.239 | <.001 | .051 | 2.897 | .004 |
|  | Comorbidity status ^h^ | .050 | 3.219 | .001 | .010 | .527 | .598 |
|  | Social media user ^f^ | .023 | 1.541 | .123 | .012 | .714 | .475 |
|  | Social media use frequency ^i^ | -.009 | -.602 | .547 | -.014 | -.782 | .434 |
| **Model 3**  Male: R^2^=.020, Adjusted R^2^=.015, *p*<0.001  Female: R^2^=.019, Adjusted R^2^=.013, *p*<0.001 | (Constant) |  | 8.596 | <.001 |  | 5.681 | <.001 |
|  | Age | -.045 | -2.144 | .032 | -.034 | -1.482 | .138 |
|  | Education level ^a^ | .023 | 1.528 | .127 | .066 | 3.768 | <.001 |
|  | Occupation ^b^ | -.026 | -1.443 | .149 | -.030 | -1.503 | .133 |
|  | Residence ^c^ | -.051 | -3.309 | .001 | -.023 | -1.268 | .205 |
|  | Administrative division ^d^ | -.007 | -.466 | .641 | -.043 | -2.469 | .014 |
|  | Marital status ^e^ | .026 | 1.365 | .172 | .048 | 2.162 | .031 |
|  | Smoking status ^f^ | .013 | .829 | .407 | .002 | .103 | .918 |
|  | Alcohol consuming ^f^ | .013 | .843 | .399 | .005 | .303 | .762 |
|  | Perceived health status ^g^ | .079 | 5.242 | <.001 | .049 | 2.758 | .006 |
|  | Comorbidity status ^h^ | .051 | 3.301 | .001 | .012 | .686 | .493 |
|  | Social media user ^f^ | .026 | 1.782 | .075 | .019 | 1.094 | .274 |
|  | Social media use frequency ^i^ | -.015 | -1.018 | .309 | -.018 | -.991 | .322 |
|  | SRHF Dhaka ^f^ | .025 | 1.666 | .096 | .012 | .712 | .477 |
|  | SRHF COVID-19 infected country ^f^ | -.007 | -.505 | .614 | .000 | -.020 | .984 |
|  | SCI: Social media ^h^ | .067 | 4.369 | <.001 | .043 | 2.446 | .014 |
|  | SCI: YouTube ^h^ | -.008 | -.476 | .634 | .033 | 1.767 | .077 |
|  | SCI: Newspaper ^h^ | -.023 | -1.360 | .174 | -.022 | -1.144 | .253 |
|  | SCI: Television ^h^ | .016 | .969 | .333 | .014 | .765 | .444 |
|  | SCI: Health website ^h^ | -.006 | -.398 | .691 | -.012 | -.677 | .498 |
|  | SCI: Other sources ^h^ | -.008 | -.534 | .593 | -.059 | -3.306 | .001 |
| β, Standardized Regression Coefficient; SBHF, Someone returns to home from; SCI, Source of COVID-19 information.  ^a^1=Lower, 2=Higher; ^b^1=Unemployed, 2=Employed, 3=Retried, 4=Housewife, 5=Student; ^c^1=Village, 2=Upzilla town, 3=District-level town, 4=Divisional city; ^d^1=Barishal, 2=Chittagong, 3=Dhaka, 4=Khulna, 5=Mymensingh, 6=Rajshahi, 7=Rangpur, 8=Sylhet; ^e^1=Single, 2=Married, 3=Divorced/ widowed/ others; ^f^1=Yes, 2=No; ^g^1=Very good, 2=Acceptable, 3=Poor, 4=Very poor; ^h^1=No, 2=Yes; ^i^1=More than 4 day/week, 2=2/3 days a week, 3=Everyday, 4=Several times a day. | | | | | | | |

**Supplementary Table 2.** Education level-based hierarchical regression analysis predicting fear of COVID-19

| **Models** | | **Lower education** | | | **Higher education** | | |
| --- | --- | --- | --- | --- | --- | --- | --- |
|  |  | ***β*** | ***t*** | ***p*** | ***β*** | ***t*** | ***p*** |
| **Model 1**  LE: R^2^=.030, Adjusted R^2^=.025, *p*<0.001  HE: R^2^=.055, Adjusted R^2^=.054, *p*<0.001 | (Constant) |  | 16.090 | <.001 |  | 33.512 | <.001 |
|  | Age | .059 | 1.343 | .180 | -.049 | -3.066 | .002 |
|  | Gender ^a^ | .164 | 5.111 | <.001 | .227 | 18.461 | <.001 |
|  | Occupation ^b^ | -.042 | -1.115 | .265 | -.024 | -1.658 | .097 |
|  | Residence ^c^ | -.050 | -1.615 | .107 | -.039 | -3.207 | .001 |
|  | Administrative division ^d^ | -.058 | -1.957 | .051 | -.018 | -1.531 | .126 |
|  | Marital status ^e^ | -.023 | -.522 | .602 | .050 | 3.346 | .001 |
| **Model 2**  LE: R^2^=.041, Adjusted R^2^=.031, *p*<0.001  HE: R^2^=.062, Adjusted R^2^=.060, *p*<0.001 | (Constant) |  | 6.321 | <.001 |  | 11.875 | <.001 |
|  | Age | .030 | .654 | .513 | -.060 | -3.684 | <.001 |
|  | Gender ^a^ | .147 | 4.391 | <.001 | .220 | 17.053 | <.001 |
|  | Occupation ^b^ | -.056 | -1.460 | .145 | -.027 | -1.859 | .063 |
|  | Residence ^c^ | -.038 | -1.221 | .223 | -.038 | -3.141 | .002 |
|  | Administrative division ^d^ | -.054 | -1.813 | .070 | -.017 | -1.482 | .138 |
|  | Marital status ^e^ | -.031 | -.701 | .484 | .049 | 3.320 | .001 |
|  | Smoking status ^f^ | .042 | 1.238 | .216 | .006 | .482 | .630 |
|  | Alcohol consuming ^f^ | .034 | 1.083 | .279 | .007 | .538 | .591 |
|  | Perceived health status ^g^ | .038 | 1.224 | .221 | .069 | 5.700 | <.001 |
|  | Comorbidity status ^h^ | .055 | 1.668 | .096 | .031 | 2.480 | .013 |
|  | Social media user ^f^ | .012 | .396 | .692 | .018 | 1.512 | .130 |
|  | Social media use frequency ^i^ | -.054 | -1.751 | .080 | -.002 | -.206 | .837 |
| **Model 3**  LE: R^2^=.047, Adjusted R^2^=.030, *p*<0.001  HE: R^2^=.067, Adjusted R^2^=.064, *p*<0.001 | (Constant) |  | 4.882 | <.001 |  | 9.262 | <.001 |
|  | Age | .027 | .590 | .555 | -.057 | -3.507 | <.001 |
|  | Gender ^a^ | .142 | 4.192 | <.001 | .216 | 16.558 | <.001 |
|  | Occupation ^b^ | -.061 | -1.584 | .113 | -.027 | -1.896 | .058 |
|  | Residence ^c^ | -.033 | -1.034 | .301 | -.039 | -3.101 | .002 |
|  | Administrative division ^d^ | -.052 | -1.757 | .079 | -.016 | -1.320 | .187 |
|  | Marital status ^e^ | -.030 | -.665 | .506 | .047 | 3.144 | .002 |
|  | Smoking status ^f^ | .042 | 1.252 | .211 | .007 | .503 | .615 |
|  | Alcohol consuming ^f^ | .032 | 1.010 | .313 | .006 | .464 | .643 |
|  | Perceived health status ^g^ | .037 | 1.186 | .236 | .067 | 5.536 | <.001 |
|  | Comorbidity status ^h^ | .054 | 1.642 | .101 | .032 | 2.621 | .009 |
|  | Social media user ^f^ | .025 | .790 | .430 | .018 | 1.544 | .123 |
|  | Social media use frequency ^i^ | -.052 | -1.645 | .100 | -.008 | -.678 | .498 |
|  | SRHF Dhaka ^f^ | -.004 | -.125 | .900 | .023 | 1.934 | .053 |
|  | SRHF COVID-19 infected country ^f^ | .001 | .043 | .966 | -.005 | -.419 | .675 |
|  | SCI: Social media ^h^ | .031 | .962 | .336 | .059 | 4.929 | <.001 |
|  | SCI: YouTube ^h^ | -.034 | -1.057 | .291 | .015 | 1.195 | .232 |
|  | SCI: Newspaper ^h^ | .001 | .033 | .974 | -.027 | -2.018 | .044 |
|  | SCI: Television ^h^ | .001 | .024 | .981 | .016 | 1.276 | .202 |
|  | SCI: Health website ^h^ | -.001 | -.032 | .975 | -.009 | -.702 | .483 |
|  | SCI: Other sources ^h^ | -.061 | -1.966 | .050 | -.022 | -1.806 | .071 |
| β, Standardized Regression Coefficient; SBHF, Someone returns to home from; SCI, Source of COVID-19 information. LE, Lower Education; HE, Higher education.  ^a^1=Male, 2=Female; ^b^1=Unemployed, 2=Employed, 3=Retried, 4=Housewife, 5=Student; ^c^1=Village, 2=Upzilla town, 3=District-level town, 4=Divisional city; ^d^1=Barishal, 2=Chittagong, 3=Dhaka, 4=Khulna, 5=Mymensingh, 6=Rajshahi, 7=Rangpur, 8=Sylhet; ^e^1=Single, 2=Married, 3=Divorced/ widowed/ others; ^f^1=Yes, 2=No; ^g^1=Very good, 2=Acceptable, 3=Poor, 4=Very poor; ^h^1=No, 2=Yes; ^i^1=More than 4 day/week, 2=2/3 days a week, 3=Everyday, 4=Several times a day. | | | | | | | |
